# Supplementary material for: Waist circumference and glycaemia are strong predictors of progression to diabetes in individuals with prediabetes in sub-Saharan Africa: 4-year prospective cohort study in Malawi
Source: PLOS Glob Public Health. 2023 Sep 27;3(9):e0001263. doi: 10.1371/journal.pgph.0001263 (PMC10529551; doi:10.1371/journal.pgph.0001263)
Supplement: S1 Table — (DOCX) [file pgph.0001263.s001.docx]

**S1 Table** Comparison between participants who were found and not found at follow up

|  | Not Followed up  (N=199) | Followed up  (N=175) | P Value |
| --- | --- | --- | --- |
| Location: Rural (%) | 38(25) | 112(75) | <0.001 |
| Urban (%) | 136(68) | 63(32) |  |
| Sex: Male (%) | 72(57) | 54(53) | 0.66 |
| Female (%) | 102(46) | 121(54) |  |
| Age (years) | 38.3±14.4 | 48±14.7 | <0.001 |
| BMI (kg/m2 ) | 27.3±6.6 | 27.5±5.9 | 0.89 |
| Waist (cm) | 88.4±15.4 | 91.1±11.9 | 0.20 |
| Fasting plasma glucose (mmol/L) | 6.3±0.2 | 6.4±0.2 | 0.13 |
