# Supplementary material for: Waist circumference and glycaemia are strong predictors of progression to diabetes in individuals with prediabetes in sub-Saharan Africa: 4-year prospective cohort study in Malawi
Source: PLOS Glob Public Health. 2023 Sep 27;3(9):e0001263. doi: 10.1371/journal.pgph.0001263 (PMC10529551; doi:10.1371/journal.pgph.0001263)
Supplement: S2 Table — (DOCX) [file pgph.0001263.s002.docx]

**S2 Table** Longitudinal changes of risk factors in those that remit to Normal Glucose Tolerance (NGT) remained Impaired Fasting Glucose (IFG) or progressed to diabetes

|  | n | Regressed to NGT  106(60.6) | Remained as IFG  24(13.7) | Progressed to DM  45(25.7) | P-Value |
| --- | --- | --- | --- | --- | --- |
| Follow-up (years) | 175 | 4±2.6 | 4.3±0.9 | 5±1.9 | <0.001 |
| BMI | 168 | 0.3±2 | 0.2±1.9 | -0.6±3.2 | 0.115 |
| Waist Circumference (cm) | 166 | -0.1±6.5 | 1.4±5.1 | -0.6±6.8 | 0.452 |
| Waist-hip ratio | 166 | 0±0.1 | 0±0.1 | 0±0.1 | 0.484 |
| Systolic BP (mmHg) | 170 | 0.5±17.5 | 2.3±22.9 | -2.9±25.5 | 0.537 |
| Fasting Plasma Glucose (mmol/L) | 173 | -1.3±0.6 | 0±0.3 | 4.1±4.7 | <0.001 |
| Cholesterol (mmol/L) | 174 | -0.1±0.8 | 0.4±0.7 | 0.1±1.2 | 0.068 |
| Tryglycerides (mmol/L) | 173 | 0.1±0.8 | 0.2±0.9 | 0.3±1.1 | 0.665 |
| HDL-C (mmol/L) | 172 | 0.1±0.3 | 0.1±0.2 | 0.1±0.3 | 0.684 |
| LDL-C (mmol/L) | 174 | -0.4±0.6 | -0.2±0.7 | -0.5±1 | 0.141 |
