# Supplementary material for: Waist circumference and glycaemia are strong predictors of progression to diabetes in individuals with prediabetes in sub-Saharan Africa: 4-year prospective cohort study in Malawi
Source: PLOS Glob Public Health. 2023 Sep 27;3(9):e0001263. doi: 10.1371/journal.pgph.0001263 (PMC10529551; doi:10.1371/journal.pgph.0001263)
Supplement: S3 Table — (DOCX) [file pgph.0001263.s003.docx]

**S3 Table** Multivariate logistic regression of risk factors to the progression of IFG using participants from the rural area

| Variables | Model 1  Standardised logistic regression adjusted for follow-up | | Model 2  Adjusted for follow-up and age | | Model 3  Adjusted for follow-up and BMI | | Model 4  Adjusted for follow-up and waist circumference | | |
| --- | --- | --- | --- | --- | --- | --- | --- | --- | --- |
|  | **OR (95% CI)** | **P Value** | **OR (95% CI)** | **P Value** | **OR (95% CI)** | **P Value** | **OR (95% CI)** | **P Value** | |
|  |  |  |  |  |  |  |  | |  |
| Age | 1.68 (0.99 - 2.85) | 0.054 | - |  | 1.60 (0.91 – 2.80) | 0.10 | 1.40 (0.77 – 2.52) | 0.27 | |
| BMI | 1.90 (1.08 – 3.34) | 0.03 | 1.80 (1.01 – 3.17) | 0.04 | - | 0.03 | 0.53 (0.18 - 1.59) | 0.26 | |
| Waist circumference | 2.54 (1.42 – 4.53) | 0.002 | 2.36 (1.30 – 4.27) | 0.005 | 4.29 (1.43 – 12.9) | 0.009 | - |  | |
| Waist hip ratio | 2.21 (1.23 – 3.96) | 0.007 | 1.98 (1.07 – 3.66) | 0.03 | 2.14 (1.17 – 3.92) | 0.014 | 1.57 (0.81 – 3.7) | 0.18 | |
| Systolic BP | 1.40 (0.89 – 2.20) | 0.14 | 1.18 (0.71 – 1.95) | 0.53 | 1.24 (0.78 – 1.97) | 0.36 | 1.03 (0.63 – 1.70) | 0.89 | |
| Fasting plasma glucose | 1.18 (0.74 – 1.89) | 0.49 | 1.04 (0.64 – 1.70) | 0.86 | 1.21 (0.75 – 1.97) | 0.43 | 1.21 (0.73 – 1.99) | 0.47 | |
| Cholesterol | 1.52 (0.93 - 2.49) | 0.09 | 1.32 (0.77 – 2.25) | 0.31 | 1.32 (0.77 – 2.24) | 0.31 | 1.27 (0.74 – 2.20) | 0.39 | |
| Triglycerides | 1.25 (0.76 – 2.05) | 0.38 | 1.09 (0.64 – 1.85) | 0.76 | 1.06 (0.61 – 1.85) | 0.82 | 0.88 (0.49 – 1.59) | 0.68 | |
| HDL-Cholesterol | 1.14 (0.72 – 1.80) | 0.58 | 1.16 (0.72 – 1.86) | 0.54 | 1.18 (0.72 – 1.93) | 0.51 | 1.31 (0.76 – 2.19) | 0.35 | |
| LDL- Cholesterol | 2.03 (1.19 – 3.48) | 0.01 | 1.84 (1.04 – 3.26) | 0.03 | 1.82 (1.03 – 3.20) | 0.04 | 1.74 (0.97 – 3.11) | 0.06 | |
| Sex | 0.99 (0.37 – 2.64) | 0.98 | 1.12 (0.37 – 2.76) | 0.99 | 0.71 (0.25 – 2.03) | 0.52 | 0.83 (0.29 – 2.35) | 0.72 | |
| Wealth | 2.86 (0.64 – 12.7) | 0.17 | 2.84 (0.62 – 13.0) | 0.18 | 1.36 (0.69 – 2.69) | 0.38 | 1.95 (0.41 – 9.26) | 0.40 | |
