# Supplementary material for: Waist circumference and glycaemia are strong predictors of progression to diabetes in individuals with prediabetes in sub-Saharan Africa: 4-year prospective cohort study in Malawi
Source: PLOS Glob Public Health. 2023 Sep 27;3(9):e0001263. doi: 10.1371/journal.pgph.0001263 (PMC10529551; doi:10.1371/journal.pgph.0001263)
Supplement: S4 Table — (DOCX) [file pgph.0001263.s004.docx]

**S4 Table** Diagnostic accuracy of waist circumference and baseline fasting glucose concentration as a screening tool for predicting the progression of diabetes in people with pre-diabetes

| Test | Diagnostic Accuracy (%) |
| --- | --- |
| Sensitivity | 31.11 |
| Specificity | 93.75 |
| Positive predictive value | 63.64 |
| Negative predictive value | 79.47 |
| Area under ROC curve | 78.60 |
