# Supplementary material for: Waist circumference and glycaemia are strong predictors of progression to diabetes in individuals with prediabetes in sub-Saharan Africa: 4-year prospective cohort study in Malawi
Source: PLOS Glob Public Health. 2023 Sep 27;3(9):e0001263. doi: 10.1371/journal.pgph.0001263 (PMC10529551; doi:10.1371/journal.pgph.0001263)
Supplement: Attachment — Submitted filename: Response to Reviewers .docx [file pgph.0001263.s005.docx]

**Response to Reviewers**

Thank you for reviewing the manuscript titled: Waist circumference and glycaemia are strong predictors of progression to diabetes in individuals with prediabetes in sub-Saharan Africa: 4-year prospective cohort study in Malawi. Below are the responses to the reviewers.

**Reviewer #1**

1 - Abstract
Conclusions: State only what your study found; do not include extraneous information not backed up by the results.

Thank you for this comment. We have removed the incidences of diabetes found in other settings from the abstract section of the manuscript.

2 - Discussion
Compare and contrast your study with others in the most relevant world literature, particularly the recent literature.

We have compared and contrasted the findings of our study to other research done worldwide. We found that the incidence rate of diabetes in people with IFG in Malawi is higher than that seen in Europe or the USA but similar to that in India. In sub-Saharan Africa, data on prediabetes and its progression is very limited, and this study offers vital insights into the future burden of the disease in the area.

3 - What new information is sufficient to modify existing clinical practice?

The new information found in the study is that the incidence rates of diabetes in people with impaired fasting glucose in Malawi (and possibly in other low-income sub-Saharan countries) are higher than that seen in Europe or the USA but similar to that seen in India. We also found that waist circumference and the initial blood glucose concentration are the strongest predictors of progression. A simple map with probabilities of progression to diabetes based on waist circumference and fasting plasma glucose could be used to identify those at risk of progression in Sub-Saharan Africa.

4 -What are the conclusions and implications for current practice, and particularly for future research that may have a significant impact on clinical decisions?

The main conclusion from our study is that a simple contour map with probabilities of progression to diabetes based on waist circumference and FPG could be used in a clinical setting to identify those at risk of progression. Glucose tests are now available worldwide, so this map is a practical and pragmatic tool that health officials could use to communicate the likelihood of patients with IFG to progress to diabetes using simple clinical features without expensive tests or age, which is not always accurate. Further tests are needed to validate this map in this population and other SSA populations.

5 - How can this study affect public policies related to health?

Several studies that have reported the prevalence of diabetes and prediabetes in sub-Saharan Africa, but only a few cohort studies have been done to determine the incidence of these conditions in the region. We did not find any studies that have determined the progression rate of diabetes in people with impaired fasting glucose in sub-Saharan Africa.

6 - What does this study add to the literature?

Thank you for this question. Prediabetes is a transition state to diabetes and offers a unique window to intervene before the complete development of diabetes. The rate of progression in prediabetes individuals has been shown to vary between different study populations. However, data on the rate in sub-Saharan Africa is limited. This study aimed to determine the incidence of diabetes in Malawi.

The study found that the incidence rates of diabetes in people with impaired fasting glucose in Malawi are higher than that seen in Europe or the USA but similar to that seen in India.

Waist circumference and the initial blood glucose concentration are the strongest predictors of progression. A simple map with probabilities of progression to diabetes based on waist circumference and fasting plasma glucose could be used to identify those at risk of progression in Sub-Saharan Africa.

7 - At the end of the Discussion, under the subheading “Limitations,” review the limitations of your study.

The study’s limitations are stated under the strengths and limitations section of the discussion. We have separated these subheadings, and the limitations section discusses the limitations of the study in detail.

8 - At the end of the limitations, under the subheading “Future directions”.

Thank you for this suggestion. We have included future directions in the implications section of the discussion.

9 - Conclusion
Take special care to draw your conclusions only from your results and verify that your conclusions are firmly supported by your data

Thank you for this comment. We have ensured that the conclusions are those derived from the findings of our current study.

10 - Table 1
I suggest include: metabolic syndrome, lipids ratio, exercise, hypertension, diabetes, smokers, liver steatosis, alcohol intake, Non-HDL cholesterol, COPD¸ Gout, Carotid artery disease, Hypothyroidism, Anemias, Drug abuse, asthma, atrial fibrillation, high-sensitivity C-reactive protein, Chronic kidney disease, Dialysis, Heart failure, Liver cirrhosis, Gastrointestinal bleeding history, Fasting glucose, Depression, Valve disorder, Blood pressure (Systolic and Diastolic), Heart rate, bpm; Hemoglobin, g/dL, mean (SD), Platelet, x 109/L, mean (SD) and medications. Family history of stroke, CAD, PAD. History of cancer.

Lipids, alcohol intake, fasting glucose, blood pressure, and family history of diabetes are included in table 1. We added physical activity to the analysis, and there was no difference in physical activity levels between those who progressed to diabetes and those who did not. People in Africa tend to be physically active, as observed in the baseline survey that 94% of people in rural and urban Malawi met the WHO recommended level of physical activity. Therefore, we did not expect physical activity to be a significant risk factor for progression in the population.

The present study is a baseline upon which our future work will build on. We did not collect data on liver steatosis, COPD¸ Gout, Carotid artery disease, Hypothyroidism, Anemias, substance abuse, asthma, atrial fibrillation, high-sensitivity C-reactive protein, dialysis, heart failure, Liver cirrhosis, Gastrointestinal bleeding history, Depression, Valve disorder; Hemoglobin, Platelet, x 109/L. Family history of stroke, CAD, PAD or History of cancer.

11– References
Include a list of abbreviations under all tables.

All abbreviations are expounded in the descriptions of all the tables.

Include a list of abbreviations before the Abstract.

We have included a list of abbreviations before the abstract, though we have not seen this in other manuscripts published by the journal. We shall leave it to the editors to decide whether to leave it or not.

12 - How was the sample size calculated?

Thank you so much for the comment. For the study, we used the Cochran formula to calculate the power of the study. Assuming a progression rate of 4% per year (based on previously published data of 15-30% over five years), the sample size of 388 followed up after approximately three years will give us around 1.1% absolute precision of annual incidence rate of diabetes among pre-diabetics with a confidence interval of 95%.

13 - Why was it made to use statistical tests?

Whilst we recruited fewer participants than anticipated, we found a similar incidence of progression to diabetes as had been found in other published research and was expected in our study (25.6%). The 175 participants recruited gave us an absolute precision of 2.07% which is adequate to enable planning for prevention and intervention programs in the region.

14- Include a table with measures of sensitivity, specificity, positive and negative predictive values, positive and negative likelihood ratios, area under the ROC curve, diagnostic odds and Youden index.

Thank you for this suggestion. The area under the receiver operator characteristics (ROC) curves is included in table 3, together with the pseudo r2 and the 95% confidence intervals, illustrating the different models’ performance.

**Reviewer #2**

1- Table 1 and 2 may be given with significant values in bold.

Thank you for this. We have edited table 1 to make it uniform with the other tables

2- Cite a reference for the article “Blood pressure and triglycerides are highly correlated with obesity. Several studies also found that higher triglycerides and blood pressure at baseline in those with prediabetes were associated with progression to diabetes”

We have included the citations for the two statements written in the discussion section of the manuscript.

Authors unpublished observation can’t be cited.

We have removed our unpublished observation in the discussion, which stated that in a study of 800 participants in Malawi, we found only ten per cent of the participants with prediabetes had isolated impaired glucose tolerance

While submitting authors may insert line numbers.

This is noted.
