# Supplementary material for: Waist circumference and glycaemia are strong predictors of progression to diabetes in individuals with prediabetes in sub-Saharan Africa: 4-year prospective cohort study in Malawi
Source: PLOS Glob Public Health. 2023 Sep 27;3(9):e0001263. doi: 10.1371/journal.pgph.0001263 (PMC10529551; doi:10.1371/journal.pgph.0001263)
Supplement: Attachment — Submitted filename: Response to reviewers.docx [file pgph.0001263.s006.docx]

**Response to Reviewers**

Thank you for reviewing the manuscript titled: Waist circumference and glycaemia are strong predictors of progression to diabetes in individuals with prediabetes in sub-Saharan Africa: 4-year prospective cohort study in Malawi. Below are the responses to the reviewers.

1 - Include a table with measures of sensitivity, specificity, positive and negative predictive values, positive and negative likelihood ratios, area under the ROC curve, diagnostic odds and Youden index.

Thank you for this comment. We have added the table with the diagnostic accuracy of the model using waist circumference and fasting glucose to predict progression to diabetes in the supplementary section of the manuscript.

2 - The subtopic Strengths can be added to Implications and future directions

Thank you for this comment. We have added the paragraph discussing the strengths of the study to the section on implications and future directions.
