# Supplementary material for: Waist circumference and glycaemia are strong predictors of progression to diabetes in individuals with prediabetes in sub-Saharan Africa: 4-year prospective cohort study in Malawi
Source: PLOS Glob Public Health. 2023 Sep 27;3(9):e0001263. doi: 10.1371/journal.pgph.0001263 (PMC10529551; doi:10.1371/journal.pgph.0001263)
Supplement: Attachment — Submitted filename: Responses to reviewers comments.docx [file pgph.0001263.s007.docx]

REVIEWERS COMMENTS

**Waist circumference and glycaemia are strong predictors of progression to diabetes in individuals with prediabetes in sub-Saharan Africa: 4-year prospective cohort study in Malawi**

Journal: Plos Global Public Health

Reviewer: 3

Comments to the Author
Reviewer #3: Title: Waist circumference and glycaemia are strong predictors of progression to diabetes in individuals with prediabetes in sub Saharan Africa: 4-year prospective cohort study in Malawi
Comment: the title is too long, it also gives an implication about the findings of the study not the objective. We can not say “Waist circumference and glycaemia are strong predictors of progression to diabetes” This means we already know the answer to our research question in advance. Also, the title includes two settings “Sub-Saharan African” and “Malawi”. The two are different and cannot represent each other.
Authors need to change the title.

Thank you for the comment. We have changed the title to “Incidence and predictors of progression to diabetes in Malawi: a 4-year prospective cohort study of people with impaired fasting glucose”

Abstract
Results:
• “The predictors of progression were higher; age (odds ratio [OR] 1.48, 95% CI 1.01-2.19, P=0.046), BMI (OR 1.98, 95% CI 1.34-2.94, P=0.001), waist circumference (OR 2.50, 95% CI 1.60-3.9...”
Comment: I find this sentence too long and unclear to me.

We have removed the 95% CI data from each predictor. The sentence is now “The predictors of progression were higher; age (odds ratio [OR] 1.48, P=0.046), BMI (OR 1.98, P=0.001), waist circumference (OR 2.50,P<0.001), waist-hip ratio (OR 1.40, P=0.03), systolic blood pressure (OR 1.56, P=0.01), fasting plasma glucose (OR 1.53, P=0.01), cholesterol (OR 1.44, P=0.05) and low-density lipoprotein cholesterol (OR 1.80, P=0.002).”

Conclusions:
• …and in predictors of progression are like those seen in other populations”
Comment: This sentence needs to be rephrased

Thank you for the observation. We have removed the misplaced word “in”. The sentence now reads, “The incidence of diabetes in people with IFG is high in Malawi and predictors of progression are like those seen in other populations”.

• A simple chart with probabilities of progression to diabetes based on waist circumference and fasting plasma glucose could be used to identify those at risk of progression in clinical settings in sub-Saharan Africa.
Comment: This is not a conclusion. It could be a recommendation.

Thank you for your comment. We have changed the statement to “Our data also suggests that a simple chart with probabilities of progression to diabetes based on waist circumference and fasting plasma glucose could be used to identify those at risk of progression in clinical settings in sub-Saharan Africa.”

Introduction:
In this section, the authors provided on information on diabetes in Malawi per se, the country where study was conducted. All information was on SSA and this could not be representative of the country.

There are no papers that have looked at the incidence of diabetes in Malawi that we could site and discuss. We have clarified this in the introduction by adding “Few studies have determined the incidence rate and the modifiable risk factors that predispose to the development of diabetes amongst the population with prediabetes in SSA, with no studies having being conducted in Malawi”

Also, the last paragraph on diagnosis of prediabetes is too irrelevant. Authors would better provide definition of prediabetes and its prevalence in similar contexts and different age groups.

The other two reviewers have found this information useful. We also feel that this explains why we are looking at progression to diabetes in a group with impaired fasting glycaemia rather than a group with prediabetes, as is done in studies in other countries. We will thus leave it to the editor to determine whether this paragraph should be removed.

Methods:
• “We used the Cochran formula to calculate the power of the study.”
Comment: It is unclear to me what authors meant by the sentence.

Thank you for the comment. We have simplified the paragraph to make it clearer. The new paragraph is:

“We used the Cochran formula to find the margin of error in our estimate of diabetes incidence compared to the actual value. Assuming a prevalence of 4% conversion to diabetes per year, 389 subjects would give us a 1.1% absolute precision for a 95% confidence level. As only 175 subjects could be recruited, we could estimate the prevalence of diabetes conversion of 4% per year with a margin of error of 2.9% based on the width of the 95% confidence intervals.

”

Calculation of the margin of error was done to determine the precision of our results. The margin of error or the level of precision represents the maximum expected difference between the sample estimate and the true population value. For categorical data, 5% level of precision is acceptable, and for continuous data, 3% margin of error is acceptable (Krejcie & Morgan 1970).

The Cochran formula is:

E = sqrt((Z^2^ * p * (1 - p)) / n)

Where:

n is the sample size

Z is the z-score corresponding to the desired level of confidence

p is the estimated proportion or expected prevalence

E is the margin of error of the absolute precision

• “We combined self-reported physical exercise duration (minutes) and intensity (pre-coded activities, grouped into high exertion, low exertion, or sedentary) in the previous week (both at work and during leisure time) to generate average metabolic equivalent of task (MET) data per day, and categorised these into whether or not the WHO recommendations of at least 600 Total Physical Activity MET minutes per week were met”.
Comment: I am not sure how combining the duration and intensity of physical exercise can generate the MET? Is there a reference for that?

The Metabolic Equivalents (METs) is the ratio of a person’s working metabolic rate relative to the resting metabolic rate and is commonly used to express the intensity of physical activities. One MET is defined as the energy cost of sitting quietly and is equivalent to a caloric consumption of 1 kcal/kg/hour. It is estimated that compared to sitting quietly, a person’s caloric consumption is four times as high when being moderately active, and eight times as high when being vigorously active.

We have added the WHO. Global physical activity questionnaire (GPAQ) and analysis guide citation which was used to quantify levels of physical activity in this study.

Also this part: categorised these into whether or not the WHO recommendations of at least 600 Total Physical Activity MET minutes per week were met” is totally unclear

For the calculation of a categorical indicator of a populations physical activity, the total time spent in physical activity during a typical week are taken into account. One of the WHO recommendations on physical activity for health is that throughout a week, including activity for work, during transportation and leisure time, adults should do at least an equivalent combination of moderate and vigorous-intensity physical activity achieving at least 600 MET minutes.

We have added the method and included the appropriate references.

• …a trained nurse who also provided HIV screening using a rapid diagnostic test
Comment: In the Biochemical analysis it is not clear to me why was HIV screening done?

HIV screening was done because Malawi has a high prevalence of HIV/AIDS, and the role of this disease in the rise of the burden of diabetes or progression of intermediate hyperglycaemia in the country and region was not known.

• …the participant had since received a diagnosis of diabetes by health personnel”
Comment: Since when?

All participants followed up did not have diabetes at recruitment. Diagnosis of diabetes will be from the time of the baseline survey.

Statistical analysis
“We only had diagnosis data on 29% of those who progressed to diabetes. 71% were identified at follow-up. Thus exact time to progression from baseline is unknown. Therefore, we used logistic regression rather than survival analysis to interpret our data.”
Comment: This sentence is unclear to me. Please rephrase for better explanation.

The phrase has been changed “We only had diagnosis information for 29% of people who developed diabetes. The remaining 71% were identified during follow-up. Therefore, we don't know the exact time it took for them to progress from the starting point. Because of this, we used logistic regression instead of survival analysis to analyze our data”.

Results:
• “There were no significant differences in changes of clinical characteristics between those who remitted to normal glucose tolerance remained IFG or progressed to diabetes in the study period, apart from the change in age…”
Comment: What do authors mean by clinical characteristics? Especially age is not a clinical feature it is a demographic characteristic.

Thank you for this. We have removed the term clinical and the sentence now reads “There were no significant differences in changes of characteristics between those who remitted to normal glucose tolerance remained IFG or progressed to diabetes in the study period, apart from the change in age, which reflects the differences in the follow-up period”.

• In models adjusted for both age and follow-up...
Comment: why were the models adjusted for follow up period? Was it not the same for all participants? And if not why?

The baseline survey was conducted between 2012 to 2016 and the follow up was between 2018 to 2020. Participant follow up period varied depending on whether they were recruited at the start or the end of the baseline survey.

Discussion:
• In this section the authors are assuming that their study findings can be generalized to SSA inhabitants while the truth is their sample is not even representative of Malawi, one country in the region given that sample is withdrawn from one rural and one urban settings. This is actually a limitation in the study.

Thank you for this observation. Indeed Sub Saharan Africa is heterogenous and results from one country can not be used to generalise for the rest of the continent. The findings do offer however offer insight into a specific region specified by the International Diabetes Federation with very limited data.

• Also in the discussion, authors are in general repeating the findings in the “Results” section without providing any possible explanation for them. For example, they mentioned that unlike what is known in evidence: “we found that the relationship apparent in univariate analysis was lost when adjusted for BMI or waist circumference. In contrast to most studies, we observed that the association between age and incident diabetes was weak and no longer significant after adjustment for BMI or waist circumference”.
I would have expected them to try to find an explanation for that especially that in the introduction section they indicated one of the reasons for the study is that current evidence on factors for progression of prediabetes to diabetes in other countries can not be generalized to SSA.

Thank you for this observation. The relationship between age and incidence of diabetes was weak and no longer significant after adjustment of other factors. This was similar to what was observed in the many studies including the Diabetes Prevention Program. We have added this in the discussion.

• The Diabetes Prevention Program trial demonstrated that intensive lifestyle intervention, to a lesser extent, metformin use reduced the risk of diabetes progression in high-risk adults (5, 31).
Comment: I am not sure what is the relevance of those few lines where they are placed.

We have removed this sentence from the paragraph

• Strengths of the study cannot fall under the “ Implications and future directions” section
• In the limitations section, the authors mentioned that :
“However, this is the first information on the progression from intermediate hyperglycaemia in sub-Saharan Africa and so it will inform future planning in the region”.
Comment: I totally disagree with this conclusion about informing the whole region

We have rephrased the sentence to focus on Malawi and stated “However, this is the first information on the progression from intermediate hyperglycaemia in Malawi and so it will inform future planning in the country and possibly othercountries in sub-Saharan Africa.”

• “Our data suggest that a simple contour map with probabilities of progression to diabetes based on waist circumference and FPG could be used in a clinical setting to identify those at risk of progression”
Comment: I am not convinced that this map would be effective if based on investigations
in a clinical setting. How about people who do not visit clinics or live in hard-to-reach
areas?

True, the chart has potential utility in a community setting, and could possibly be used by health surveillance officers who are active in the communities to identify people who are at risk of developing diabetes. However there are very few programs in Malawi where fasting blood glucose are measured in a community setting.

All in all, the discussion section is messed up and needs to be refurbished and more evidence needs to be added to explain study findings. Recommendation that can overcome the study limitations should be included to guide future research.

We have taken on board your comments and have made revisions to the manuscripts. We have also added the following paragraph listing recommendations that can overcome the study limitations to guide future research “Research needs to be done to understand how to improve retention of patients in studies in sub-Saharan Africa or follow up people at risk of diabetes or those with the disease. This could involve developing national registers or regular record collection of people with prediabetes or diabetes and qualitative work to understand the barriers and facilitators to retaining people in studies.”
